# Supplementary material for: An analysis of production efficiency in China’s real estate industry based on a two-stage DEA model
Source: PLoS One. 2024 Oct 24;19(10):e0311174. doi: 10.1371/journal.pone.0311174 (PMC11500891; doi:10.1371/journal.pone.0311174)
Supplement: Attachment — Submitted filename: Response to Reviewers.docx [file pone.0311174.s003.docx]

**Response to Reviewers**

**Dear reviewer:**

**I hope all is well when you read the letter!**

**Thank you very much for taking the time to review this manuscript. We have made the following reflections or explanations for your questions.**

**Please find the detailed responses below and the corresponding revisions in the re-submitted files.**

**Jiening Meng**

**From the ancient capital of Luoyang**

**April 21, 2024**

Review Comments to the Author

Reviewer #1:

1. The literature review should be focused on the similar study in how to analyze the total factor productivity in different countries, regions, industries. However, no work on this area.

Indeed, there are such issues in the original paper, but when we use the DEA Malmquist index model to decompose the total factor productivity (TFP) of the real estate industry into: pure technical efficiency, technological progress efficiency, and scale efficiency, we find that the improvements in technological progress efficiency are the main driver of TFP growth in the real estate industry across 35 major and medium-sized cities in China. We believe that this offers little significance in addressing current issues such as the lack of liquidity and severe regional disparities in China's real estate market. What we should consider now is: Why is there such a large disparity in the production efficiency of the real estate industry across different regions in China? What factors are limiting the improvement of production efficiency in China's real estate industry?

Considering the recent real estate investment and sales conditions in different regions of China, this paper has opted to utilize a two-stage DEA model to measure the production efficiency of the real estate industry in 31 provinces, municipalities, and autonomous regions of China (due to data availability constraints), and to evaluate their resource utilization during the development and sales stages. The results were enlightening.

Additionally, in the updated literature review, we have shifted away from merely listing literature by Chinese scholars or studies related to the production efficiency of the Chinese real estate industry. Instead, we have structured it around the thematic thread of "distinct issues in real estate markets - spatial discrepancies in real estate prices - regional variations in real estate production efficiency," summarizing relevant achievements and shortcomings. Furthermore, in the section discussing models and methods, a brief overview of relevant issues concerning efficiency estimation is provided when discussing why a two-stage DEA model was chosen and features of its analysis method.

2. Regarding the methods, I think the authors should focus on the why the DEA Malmquist index model suits this study, rather than show what is the DEA Malmquist index model by 3-4 pages.

After 2014, the most significant challenge facing the development of China's real estate industry has been the phenomenon of regional differentiation in real estate markets. To better understand the utilization of real estate resources in different regions and reveal the reasons for their disparities, this paper has switched to employing a two-stage DEA model to measure the production efficiency of the real estate industry in various regions and to thoroughly assess their resource utilization during the development and sales stages. The analysis results indicate that the low efficiency and significant gap during the sales stage are the primary reasons for the regional differences in the production efficiency of the Chinese real estate industry, which holds crucial significance for effectively addressing related issues.

Given the adoption of a new method, the two-stage DEA model, all explanations regarding the DEA-Malmquist index model from the original paper have been omitted.

3. Data source is the base of this study. However, how to obtain the relevant data is not clarified.

We have added data sources in the revised draft:

The primary data sources are the Wind database, with additional data on the real estate market and influencing factors derived from the "China Real Estate Yearbook" and "China Statistical Yearbook" (2015-2023). Production efficiency is calculated using Dearun.

Reviewer #2:

1. The DEA Malmquist index model measures production efficiency. The selection of input factors is very important. How to select and decide?

Neoclassical economics categorizes all production factors into four types: land, capital, labor, and entrepreneurial talent. Following this line and considering the availability of relevant statistical data in China, this paper focuses on analyzing the quantities of people, land, and money utilized in the processes of real estate development and sales.

2. The real estate industry is greatly affected by overall economic and policy factors. How does this model deal with these two factors?

In the process of calculating production efficiency, we only consider the quantities of input factors and output products, as DEA generally treats the production process as a "black box." However, when analyzing the factors affecting the production efficiency of the real estate industry using the Tobit model and threshold effect model, we incorporate relevant indicators reflecting macroeconomic conditions, regional economic and social development, orientations of real estate regulation policies, and internal organizational management status of real estate development enterprises as explanatory or control variables. The regression coefficients of these variables indicate significant effects among them.

3. Generally speaking, the larger the R square, the better the fit between the model and the data. The R square in Table 5 is only between 10.8% and 12.9%, indicating that the model fit in this article is low and needs to be re-examined.

Indeed, the original paper encounters the issues you mentioned when analyzing the roles of various influencing factors using the Moderating effect model. After careful consideration, we found that efficiency values are constants between 0 and 1. Hence, when analyzing their influencing factors, a constrained dependent variable model, the Tobit model, should be utilized. However, due to the limited availability of data concerning the real estate industry in 35 major cities in China, the direct application of the Tobit model for data analysis yielded unsatisfactory results. Consequently, this paper had to substitute samples and data, gathering relevant data on the real estate industry in 31 provinces, municipalities, and autonomous regions of China from 2014 to 2022. The utilization of the Tobit model for regression improved significantly, achieving a fit of around 70%, compared to previous attempts.

4. What impact does the result of this article have on the existing "purchase restriction orders" in first-tier cities and the "destocking" policies in third- and fourth-tier cities?

The "purchase restriction orders" and "destocking" measures are primarily mentioned to illustrate that without understanding the underlying reasons for the lower production efficiency of the real estate industry in various regions, government departments tend to focus solely on formulating policies related to real estate sales. However, this approach not only fails to fundamentally alter the phenomenon of irrational allocation of real estate resources but also constitutes excessive government intervention in the operation of the real estate market. If the unreasonable utilization of resources in the real estate development or sales stage can be identified promptly, adjustments in the quantities of input factors at each stage can be made in a timely manner. By reducing the number of real estate developments and minimizing the creation of inventory, the issue of misallocation of real estate resources can be addressed at its root.
