# Supplementary material for: An analysis of production efficiency in China’s real estate industry based on a two-stage DEA model
Source: PLoS One. 2024 Oct 24;19(10):e0311174. doi: 10.1371/journal.pone.0311174 (PMC11500891; doi:10.1371/journal.pone.0311174)
Supplement: Attachment — Submitted filename: Response to Reviewers (Aug).docx [file pone.0311174.s004.docx]

Dear Prof Ahmad and reviewers,

I am writing on behalf of all the contributing author to express our sincere appreciation for your letter and the constructive comments provided by the reviewer regarding our article entitled "An Analysis of Production Efficiency in China's Real Estate Industry Based on a Two-Stage DEA Model" (Manuscript No.: PONE-D-23-38796R1). These comments are extremely valuable and have greatly contributed to improving our article. In response to these comments, extensive modifications have been made to the manuscript. In this revised version, all changes have been highlighted in red within the document. A point-by-point response to the editor and reviewer is provided below this letter.

Thank you very much for your time and consideration.

Best regards,

Jiening Meng

Corresponding author:

Name: Jiening Meng

E-mail: 16113105@bjtu.edu.cn

- Responds to Journal Requirements:

***Please review your reference list to ensure that it is complete and correct. If you have cited papers that have been retracted, please include the rationale for doing so in the manuscript text, or remove these references and replace them with relevant current references. Any changes to the reference list should be mentioned in the rebuttal letter that accompanies your revised manuscript. If you need to cite a retracted article, indicate the article’s retracted status in the References list and also include a citation and full reference for the retraction notice.***

We have thoroughly examined all the literature referenced in the article, and fortunately, there are no retracted articles. The citation and organization of the literature are also arranged in accordance with the journal's requirements. However, when we modified the content of section 3.1.2 based on the reviewer's suggestion, we replaced the 28th and 29th references and added a new one at the same time, resulting in an additional reference in the revised draft compared to the original.

- Responds to Additional Editor Comments:

***Based on the comments, the paper is given minor revisions. This is an opportunity for you to revise the paper addressing all the comments raised carefully and satisfactorily.***

According to all of the comments, we have revised the manuscript extensively and corrected several mistakes in our previous draft. If there are any other modifications we could make, we would like very much to modify them and we really appreciate your help. We hope that the changes we have made resolve all your concerns about the article. We are more than happy to make any further changes that will improve the paper or facilitate successful publication.

- Responds to Reviewer’s comments:

***1. If the authors have adequately addressed your comments raised in a previous round of review and you feel that this manuscript is now acceptable for publication, you may indicate that here to bypass the “Comments to the Author” section, enter your conflict of interest statement in the “Confidential to Editor” section, and submit your "Accept" recommendation.***

***Reviewer #3: (No Response)***

Thank you for reviewing our manuscript.

***2. Is the manuscript technically sound, and do the data support the conclusions?***

***The manuscript must describe a technically sound piece of scientific research with data that supports the conclusions. Experiments must have been conducted rigorously, with appropriate controls, replication, and sample sizes. The conclusions must be drawn appropriately based on the data presented.***

***Reviewer #3: Yes***

Thank you for your positive comments and valuable suggestions to improve the quality of our manuscript.

***3. Has the statistical analysis been performed appropriately and rigorously?***

***Reviewer #3: Yes***

Thank you for your positive comments and valuable suggestions to improve the quality of our manuscript.

***4. Have the authors made all data underlying the findings in their manuscript fully available?***

***The PLOS Data policy requires authors to make all data underlying the findings described in their manuscript fully available without restriction, with rare exception (please refer to the Data Availability Statement in the manuscript PDF file). The data should be provided as part of the manuscript or its supporting information, or deposited to a public repository. For example, in addition to summary statistics, the data points behind means, medians and variance measures should be available. If there are restrictions on publicly sharing data—e.g. participant privacy or use of data from a third party—those must be specified.***

***Reviewer #3: Yes***

Thank you for your positive comments and valuable suggestions to improve the quality of our manuscript.

***5. Is the manuscript presented in an intelligible fashion and written in standard English?***

***PLOS ONE does not copyedit accepted manuscripts, so the language in submitted articles must be clear, correct, and unambiguous. Any typographical or grammatical errors should be corrected at revision, so please note any specific errors here.***

***Reviewer #3: Yes***

Thank you again for your positive comments and valuable suggestions to improve the quality of our manuscript.

***6. Review Comments to the Author***

***Please use the space provided to explain your answers to the questions above. You may also include additional comments for the author, including concerns about dual publication, research ethics, or publication ethics. (Please upload your review as an attachment if it exceeds 20,000 characters)***

***Reviewer #3: This paper uses a two-stage Data Envelopment Analysis (DEA) model to calculate the production efficiency of China's real estate industry across 31 regions in 2014-2022. It uses a panel Tobit model to identify factors (the leverage ratio of companies, per capita GDP and regulatory policies) that significantly impact efficiency.***

***In Eq. 2, the methodology uses Tobit regression to explain efficiency. There are other regression methods such as truncated regression, robust-OLS regression or Papke-Wooldridge which are probably more suited method for these purposes. Adding a short discussion of these alternative ways for this type of analysis will be helpful (see Simar and Wilson 2008, Banker and Natarajan 2007 for discussion).***

Your recommendations have significantly enhanced the rigor of this article. After thoroughly examining the three articles you recommended, we now possess a more comprehensive understanding of econometric analysis methods for determining the influencing factors of restricted dependent variables, such as truncated regression. Consequently, we have made corresponding modifications to the paper. Additionally, we faithfully included the article you recommended in our list of the references list. Once again, thank you for your meticulous review.

The following are the corresponding parts of the text:

*After assessing the production efficiency of the real estate industry in different regions, this paper considers the efficiency values as dependent variables, and factors other than input-output data, reflecting the heterogeneity of the decision-making units as well as the regions, as independent variables. Drawing on insights from Amemiya, T.[28], Simar, L., and Wilson, P. W.[29], Banker, R. D., and Natarajan, R. regarding the analysis of influencing factors of restricted dependent variables, it is noted that "two-stage DEA-based procedures with OLS, ML, or even Tobit estimation in the second stage significantly outperform parametric methods"[30]. Given the practicality of computational software usage, this article employs a Tobit model to analyze the influencing factors affecting real estate production efficiency at this stage. The basic form of the model is presented in equation (2).*

***7. PLOS authors have the option to publish the peer review history of their article (what does this mean?). If published, this will include your full peer review and any attached files.***

***If you choose “no”, your identity will remain anonymous but your review may still be made public.***

***Do you want your identity to be public for this peer review? For information about this choice, including consent withdrawal, please see our Privacy Policy.***

***Reviewer #3: No***

Thank you for reviewing our manuscript.
